# Intermediate Field Coupling of Single Epitaxial Quantum Dots to Plasmonic Waveguides

**DOI:** 10.1021/acs.nanolett.3c03442

**Published:** 2023-11-02

**Authors:** Michael Seidel, Yuhui Yang, Thorsten Schumacher, Yongheng Huo, Saimon Filipe Covre da Silva, Sven Rodt, Armando Rastelli, Stephan Reitzenstein, Markus Lippitz

**Affiliations:** † Experimental Physics III, University of Bayreuth, Bayreuth 95447, Germany; ‡ Institute of Solid State Physics, Technische Universität Berlin, Berlin 10623, Germany; ¶ Institute of Semiconductor and Solid State Physics, Johannes Kepler University Linz, Altenbergerstraße 69, A-4040 Linz, Austria

**Keywords:** single-photon source, plasmonics, waveguide, quantum emitter, near-field

## Abstract

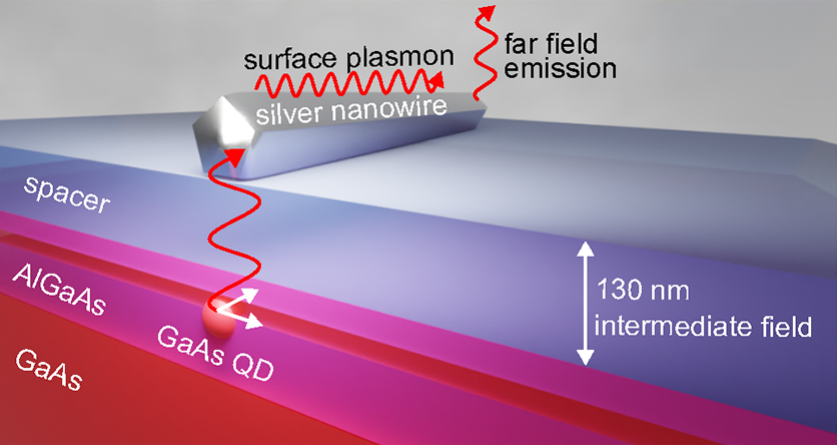

Key requirements for quantum plasmonic nanocircuits are reliable single-photon sources, high coupling efficiency to the plasmonic structures, and low propagation losses. Self-assembled epitaxially grown GaAs quantum dots are close to ideal as stable, bright, and narrowband single-photon emitters. Likewise, wet-chemically grown monocrystalline silver nanowires are among the best plasmonic waveguides. However, large propagation losses of surface plasmons on the high-index GaAs substrate prevent their direct combination. Here, we show by experiment and simulation that the best overall performance of the quantum plasmonic nanocircuit based on these building blocks is achieved in the intermediate field regime with an additional spacer layer between the quantum dot and the plasmonic waveguide. High-resolution cathodoluminescence measurements allow a precise determination of the coupling distance and support a simple analytical model to explain the overall performance. The coupling efficiency is increased up to four times by standing wave interference near the end of the waveguide.

Quantum photonics has the potential to revolutionize our world with breakthrough technologies such as quantum computing and quantum communication, for instance, using quantum dots (QDs) as single-photon emitters.^1^ Especially in terms of applications, scalability is indispensable, and integrated photonic networks are highly sought after.^2^ Plasmonic nanocircuits are a promising platform since they not only dramatically reduce circuit size but also allow light to be controlled and manipulated at a truly nanoscale level.^3−7^ Even though many electrons are involved in the surface plasmon–polariton (SPP), the quantum-optical nature is preserved.^8^

In recent years, the coupling of various quantum emitters to plasmonic waveguides has been demonstrated (for a review, see ref (9)). Sources of single plasmons have been reported at both room and liquid helium temperatures using various combinations of waveguides and emitters.^10−20^ However, for true quantum-optical operation of the circuit, high-quality sources of single photons are essential. Epitaxially grown self-assembled quantum dots possess close to ideal quantum properties as they are bright and nonblinking and have narrow line widths.^21−23^ Nonetheless, the typical approach of bringing the waveguide close to the emitter does not work, as the high refractive index of the semiconductor host induces significant damping of the SPP due to ohmic and radiative losses. Additionally, the optical properties of epitaxial quantum dots degrade with decreasing QD-to-surface distance.^24^ These issues have previously been addressed through an indirect coupling method using dielectric-plasmonic mode conversion,^16^ requiring significant nanofabrication.

Here, we address conflicting requirements in a different and much simpler way: Instead of placing the plasmonic waveguide directly on the semiconductor substrate containing quantum dots, a planar dielectric layer with a lower refractive index is used as a spacer between the semiconductor host and the plasmonic waveguide, as depicted in Figure 1a. This allows us to balance the efficiency of coupling η_in_ and propagation η_p_: a thicker spacer layer enhances the plasmon propagation length, and a thinner layer enhances the coupling efficiency between the quantum dot and the waveguide. In the following, we demonstrate that the nanocircuit performance is expected to be superior when the coupling between the emitter and waveguide occurs in the intermediate field (*kr* ≈ 1), rather than the near field (*kr* ≪ 1), where *k* and *r* denote the light wavenumber and radial distance from the emitter, respectively. Our innovative coupling scheme can convert single photons of the highest quality into propagating surface plasmons, is unexpectedly robust against emitter-waveguide displacement, and still maintains moderate device efficiency.

**Figure 1:**
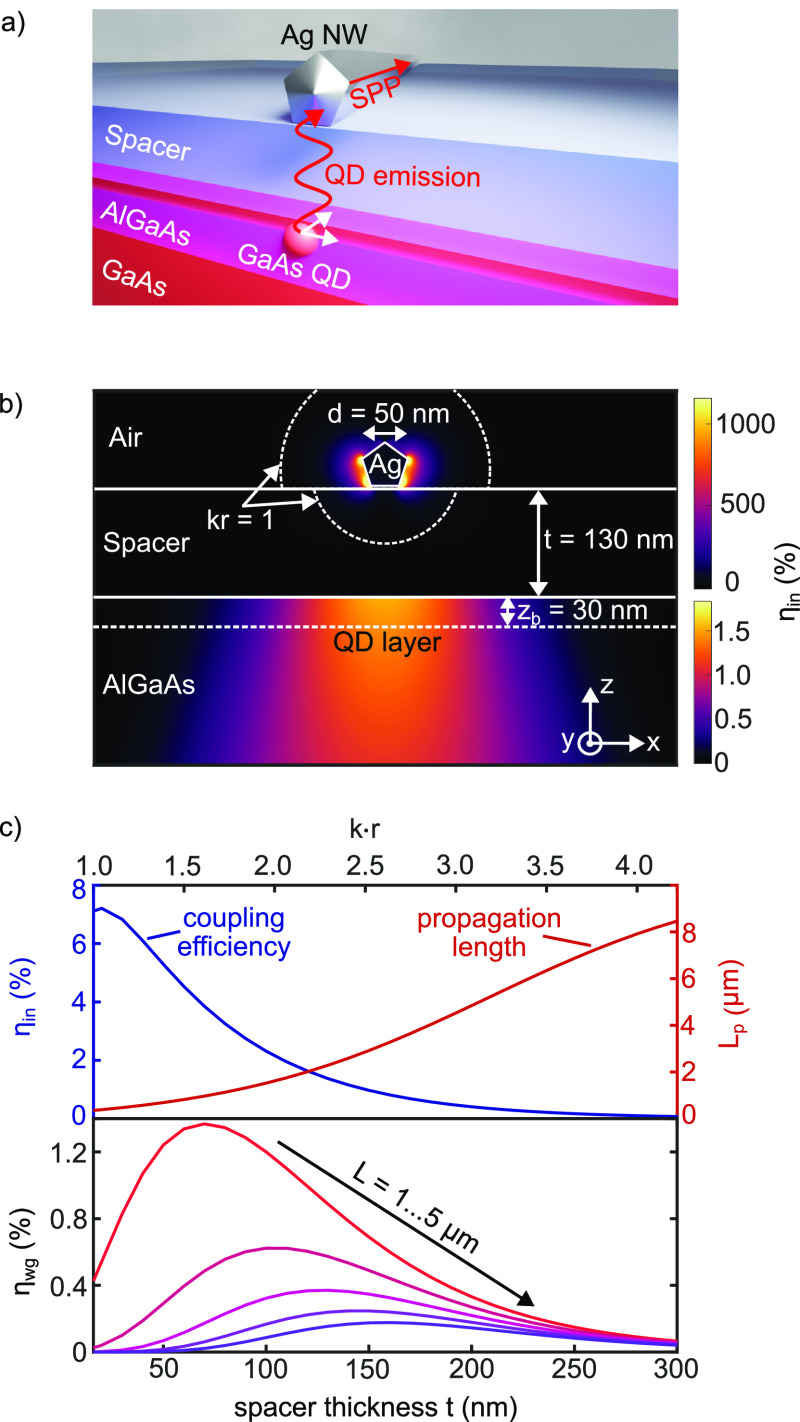
Intermediate field coupling of a quantum dot to a plasmonic waveguide. (a) Sketch of the coupling scheme: A quantum dot embedded within AlGaAs barriers radiatively couples to a silver nanowire that is separated by a capping layer and an additional dielectric spacer. (b) Spatial variation of the coupling efficiency η_in_ into the waveguide for an emitter located in the *xz*-plane and with a dipole moment in the *xy*-plane of the sample. The circle enclosing *kr* = 1 illustrates the transition between the near and far field. Note the different color scale bars for the lower and upper half spaces. (c) Upper panel: Coupling efficiency η_in_ and propagation length *L*_p_, as a function of spacer thickness *t*. The coupling efficiency is evaluated for an emitter that is centrally located beneath the nanowire in the quantum dot layer and placed at a depth of *z*_b_ = 30 nm below the spacer/AlGaAs interface. Lower panel: waveguide efficiency η_wg_ as a function of spacer thickness *t* and waveguide length *L*. Optimal performance is achieved in the intermediate field for *kr* ≳ 1.

As sketched in Figure 1a, we assume an infinitely extended waveguide in the propagation direction. Mode profiles and corresponding effective mode indices are calculated as a function of the spacer thickness *t* with Comsol Multiphysics. For all simulations, we use λ = 790 nm, the emission wavelength of our GaAs quantum dots.^25^ The chemically grown monocrystalline silver nanowire^26^ is modeled with a pentagonal cross-section (*d* = 50 nm). The refractive indices for silver (*n*_Ag_ = 0.035 + 5.49*i*) and AlGaAs (*n*_AlGaAs_ = 3.44) are taken from the literature.^27,28^ The dielectric spacer (spin-on-glass IC1-200 from Futurrex) is modeled with *n*_spacer_ = 1.41 according to the manufacturer.

To compute the coupling efficiency η_in_ for the plasmonic waveguide mode, we follow the framework in ref (29). The decay rate of the emitter into the plasmonic mode is related to the dot product between its transition dipole moment **μ** and modal field **E**^mode^. We have to consider that the transition dipole moments of our quantum dots are given by two energetically almost degenerate exciton states oriented orthogonal to each other in the sample plane. For the sake of simplicity, we assume that one of the dipole moments is oriented parallel and the other one, perpendicular to the nanowire axis. The coupling efficiency
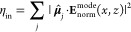
1is then obtained by incoherently adding up the two dipole moment contributions for *j* = *x*, *y*. This is done by multiplying the dipole moment unit vectors **μ̂** with the modal fields **E**_norm_^mode^(*x*, *z*), which are normalized to the emission of a dipole in homogeneous AlGaAs (see Supporting Information S1).

The coupling efficiency η_in_ for a spacer thickness of *t* = 130 nm is shown in Figure 1b as a function of the emitter position in the *xz*-plane. Note that in the simulation we place the dipole not only within the AlGaAs matrix as in the experiment but also inside the dielectric spacer and around the nanowire. Apart from the strongly confined hot spots directly at the nanowire, there is a less confined region in the semiconductor where the incoupling efficiency η_in_ does not vary much. At the quantum dot burial depth *z*_b_ ≈ 30 nm, η_in_ drops only by a factor of 2 when leaving the wire axis laterally by 125 nm. This stems from the rather loosely bound character of the waveguide mode and relaxes the required QD alignment accuracy, even though the absolute coupling efficiency is lower. Furthermore, the weak depth dependence suggests that the QD can be placed deeper in the AlGaAs without much change in the coupling efficiency. This is particularly interesting when considering that the optical properties of the QD improve rapidly with increasing burial depths.^24^

The effect of the spacer thickness on the waveguide coupling efficiency η_in_ and the propagation length *L*_p_ is shown in the top panel of Figure 1c: With increasing layer thickness, the amplitude of the waveguide mode at the quantum dot position is reduced, and therefore, the coupling efficiency decreases. Here, we evaluate the coupling efficiency for an emitter that is centered with respect to the nanowire, at a depth of *z*_b_ = 30 nm. On the other hand, the propagation length *L*_p_ of the mode is strongly increased for thicker spacers. This is due to the diminishing influence of the high-index AlGaAs, resulting in a mode that is more strongly bound to the waveguide and features less radiative losses.

For the experimental realization of our plasmonic coupling concept, we are interested in the waveguide efficiency η_wg_ = η_in_ η_p_, which also includes the propagation efficiency η_p_ = *e*^–*L*/*L*_p_^ for a waveguide of finite length *L*. Obviously, the optimal spacer thickness also depends on the waveguide length *L*, as can be seen in the lower panel of Figure 1c. Accordingly, the highest waveguide efficiency η_wg_ is achieved for short waveguides and rather thin spacers. For an experimentally meaningful nanocircuit, however, the waveguide should be longer than the spatial resolution of the optical microscope, i.e., *L* ≳ 1 μm. For such waveguide lengths, we find the optimum in the intermediate field regime at *kr* = 1.6–2.6 or *t* = 70–160 nm. Here, the spacer thickness *t* is rewritten in terms of *kr* = *k*_0_(*n*_AlGaAs_*z*_b_ + *n*_spacer_*t*) with vacuum wavenumber *k*_0_. The transition from near field to far field is also illustrated as a circle enclosing *kr* = 1 in Figure 1b.

Let us now turn to the experimental realization of such an intermediate field coupling. The sample is based on near-surface self-assembled GaAs quantum dots in AlGaAs barriers grown by molecular beam epitaxy on a GaAs substrate. These types of quantum emitters have been established as excellent single-photon sources in a variety of studies.^30−32^ For the dielectric spacer, the polysiloxane-based spin-on glass (IC1-200 Intermediate Coating, Futurrex) is spin-coated on top of the GaAs surface, resulting in a film with a thickness of *t* = (130 ± 15) nm. Chemically grown monocrystalline silver nanowires (PL-AgW100, PlasmaChem) with average widths of *d* = (50 ± 10) nm and typical lengths of a few micrometers are dispersed on top of the IC1 film. A detailed description of the sample fabrication can be found in Supporting Information S2. The random arrangement of dots and wires samples all relative orientations and coupling distances, requiring a pre-selection of potentially coupled quantum dot-nanowire pairs. Therefore, we determine the spatial arrangement by high-resolution cathodoluminescence mapping and then measure the waveguide performance by optical microscopy.

Low-temperature cathodoluminescence combines high-resolution electron microscopy with access to quantum dot emission, making it an excellent technique to specify the relative positions of quantum dots and nanowires. Our setup is described in detail in ref (33). The sample is mounted on a liquid He-flow cryostat (20 K) and excited with a focused electron beam of 20 kV acceleration voltage, which is scanned over the sample surface. As sketched in Figure 2a, the cathodoluminescence emission of each excitation spot position is collected by a spectrometer, simultaneously providing a secondary electron image and cathodoluminescence spectrum mapping with the same coordinates.

**Figure 2:**
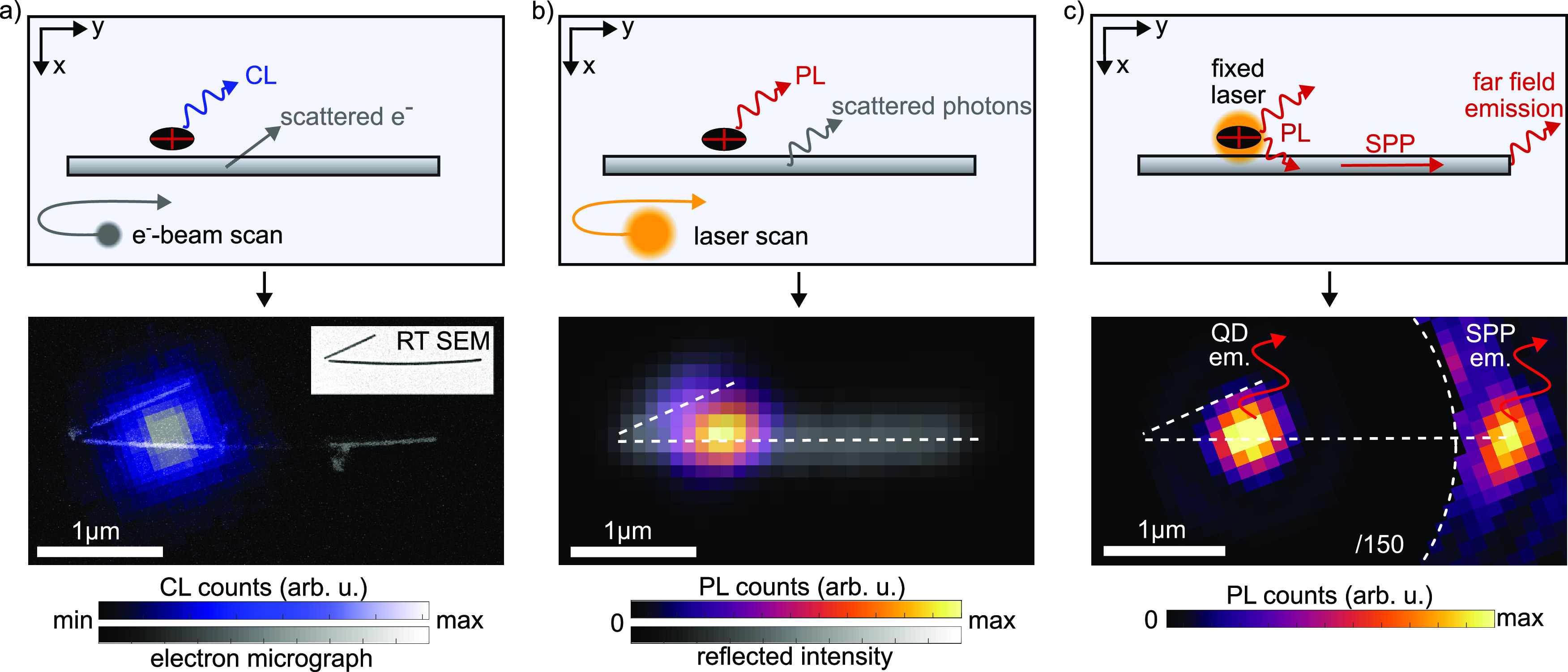
Investigation of a coupled quantum dot–nanowire system by complementary methods. (a) Raster-scanning the electron beam while detecting the cathodoluminescence and scattered electrons. The obtained cathodoluminescence image with an overlaid electron micrograph allows a precise distance measurement. The inset depicts a room temperature scanning electron micrograph of the same nanowire. (b) Sketch of confocal laser scanning imaging with corresponding photoluminescence data, overlaid with the reflection image of the structure. (c) Proof of intermediate field coupling by plasmon propagation imaging via a CCD camera and stationary excitation of the quantum dot. In the recorded image, the area around the quantum dot is software-attenuated by a factor of 150 to increase the visibility.

An example of a data set can be found in the bottom section of Figure 2a. For comparison, a room temperature scanning electron micrograph is included as an inset. Although the diameter of the electron beam is only a few nanometers, the actual size of the cathodoluminescence spot mostly results from the effective diameter of the generation volume and charge carrier diffusion.^34,35^ Hence, a two-dimensional Gaussian profile is used to fit the cathodoluminescence emission spot, allowing for precise determination of the relative lateral positions of quantum dots and nanowires with 10–30 nm accuracy. For the depicted nanosystem, the cathodoluminescence image reveals the QD positions *x*_QD_ = (77 ± 12) nm and *y*_QD_ = (685 ± 26) nm with respect to the nanowire end. However, electron beam scanning is not suitable to distinguish between direct QD emission and remote plasmon emission at the nanowire end due to the lack of spatial resolution in the detection path.

Consequently, we use an all-optical confocal microscope to demonstrate the intermediate field coupling. A fast scan mirror moves the excitation laser focus (NA = 0.9 and 635 nm wavelength) over the sample inside a closed-cycle cryostat (20 K). Different detection schemes are employed. To identify the preselected QD–nanowire system, we map the sample by photon counting and a combination of photoluminescence and reflection (Figure 2b). In order to enhance the contrast in the reflection image, the direct laser reflection is suppressed with a polarizer. Slight sample drifts during the laser scans can be neglected since this measurement is only used for identification of the nanowires and not for the coupling characterization that follows later in the text.

Intermediate field coupling is demonstrated in Figure 2c by launching and detecting plasmons: Here, the excitation laser is stationary, focused on the QD while the surrounding sample area’s luminescence is imaged onto a CCD camera. We observe clear emission from the SPP that is launched by the coupled quantum dot and scattered at the nanowire’s end. We find identical photoluminescence spectra for the direct QD emission and the outcoupled photons of the plasmon (Supporting Information S3). Emission from the short wire end is also expected but is experimentally hidden in the airy-patterned background of the direct QD emission (see Supporting Information S4).

We analyzed a total of nine QD–nanowire systems, which differ by up to a factor of 80 in the intensity ratio of the respective SPP emission *I*_spp_ and the direct QD emission *I*_qd_ (see Table S3). In the following, we extract the coupling efficiency for these nanosystems and explain this, on the first sight, large variation as an interference effect near a waveguide end.

All nine QD–nanowire systems are formed by QDs near (about 1 μm) one end of the silver waveguide. Considering typical propagation lengths in the range of a few micrometers and the small diameter of our silver nanowires, we expect substantial reflection of the SPP at the near wire end.^11^ This results in an interference |*E*|^2^ = |*E*_dir_ + *E*_refl_|^2^ of the direct surface plasmon *E*_dir_ and the reflected surface plasmon *E*_refl_ (see Figure 3a) and consequently in a position-dependent coupling efficiency. In the worst case, both fields destructively interfere with each other and no net coupling would be observed, although the emitter is close to the nanowire.

**Figure 3:**
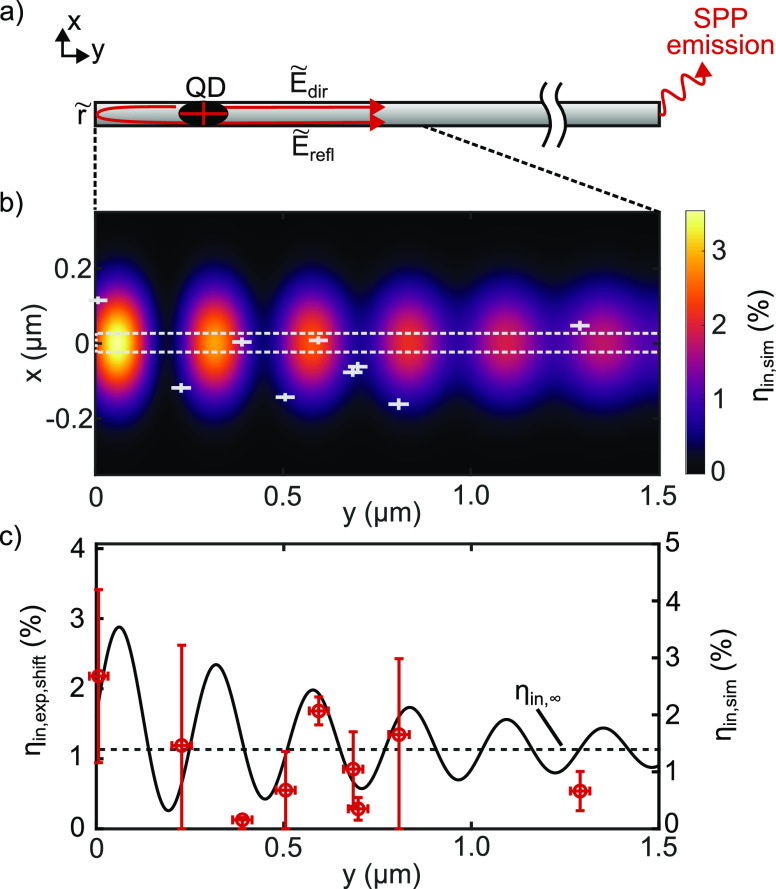
Interference caused by reflection at the waveguide end explains the spatial variation of the incoupling efficiency. (a) Schematic of the interfering SPPs, arising from nonzero reflectivity at the nanowire termination. (b) Coupling efficiency map in the sample plane at the burial depth *z*_b_ = 30 nm. The lateral quantum dot positions are displayed by gray crosses, the size of which indicates the position uncertainty from the cathodoluminescence images. The dashed horizontal lines correspond to the nanowire width. (c) Simulated coupling efficiency (black line) along the nanowire axis and experimental coupling efficiency (red circles) according to eq 3 and corrected for the offset *x*_QD_ from the nanowire axis. The dashed line represents the coupling efficiency η_in,inf_ ≈ 1.4% for a quantum dot centered below an infinitely extended wire (see Figure 1).

To model the position-dependent coupling efficiency in the *xy*-plane, we make use of reciprocity and assume a semi-infinite wire. The mode profile in the *xz*-plane is already shown in Figure 1b. We keep the *z* coordinate constant at the burial depth *z*_b_ = 30 nm to obtain the mode profile **E**^mode^(*x*). In propagation direction (*y*), we interfere the direct wave with the reflected wave, both propagating with an effective mode index *ñ*_eff_. The reflection coefficient of the wire end is also complex-valued *r̃* = *re*^*i*ϕ_r_^ with the reflection amplitude *r* and reflection phase ϕ_r_. Overall, we obtain the coupling efficiency in the *xy*-plane
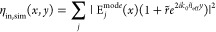
2by incoherently summing the dipole moment contributions in *j* = *x*, *y*. The resulting coupling efficiency map (Figure 3b) shows the expected oscillatory interference features that decay in interference contrast with the distance to the near waveguide end, as the amplitudes of *E*_dir_ and *E*_refl_ separate. Although measured at different waveguides, we draw all nine investigated structures in this map by overlaying the nanowire ends, which already suggests strong fluctuations in their coupling efficiency.

In the experiment, the photon rate detected at the outcoupling end of the waveguide is given by the product of the partial efficiencies for incoupling, propagation, outcoupling, and detection times the QD’s bare emission rate. At the QD position, we detect this bare rate multiplied by the QD detection efficiency. Knowing all these factors from either numerical simulations or measurements (see Supporting Information S5) allows us to calculate back to the experimentally observed incoupling efficiency η_in,exp_ at the specific QD positions relative to the waveguide.

For comparison with the interference model, it is more convenient to compare a 1D data set. Knowing the spatial mode profile, we shift the experimental QD positions to below the waveguide (*x* = 0 nm) by
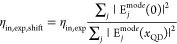
3for the offset *x*_QD_ of the respective QD via the mode profile **E**^mode^(*x*). This is the incoupling efficiency that would have been measured if the dot had been centered below the waveguide. Figure 3c compares these values to the model at *x* = 0 nm. We have fixed the reflection amplitude (*r* = 0.65) and phase (ϕ_r_ = −π/2) as well as the air-sided far-field collection efficiency ratio (η_spp,ff_/η_qd,ff_ = 17) based on our numerical simulations (see Supporting Information S5) and vary mode index, propagation length, and an overall scaling parameter. The optimal fit is achieved with a mode index of *n*_eff_ = 1.53, a propagation length *L*_p_ = 0.86 μm, and a scaling factor of 1.23. These values are already used to plot Figure 3b.

We find good agreement between our interference model and the corrected coupling efficiency, although not all individual variations of the nanosystems are taken into account. Minor differences in geometry parameters can shift the data points somewhat. In particular, imperfections such as slightly bent wires or small kinks can cause additional losses due to reflections or far field scattering but were not observed in the propagation images and are therefore neglected. Furthermore, the exact dipole moment orientations within each QD are unknown to us, which only affect QDs located far away from the nanowire axis (see Supporting Information S6). The resulting uncertainty can be quantified and is included in the error bars for the experimental coupling efficiency. In addition, the error bars comprise the uncertainty arising from the QD-SPP emission ratio extraction, and the uncertainty in the lateral QD position determination.

Nevertheless, the fit parameters consistently lie within a realistic range. For the mode index, we expect values ranging from *n*_eff_ = 1.5–1.7 from numerical simulations, depending on the details of the chosen geometry. The propagation length in the finite element simulation (see Figure 1c) is approximately *L*_p_ = 2–3 μm, around three times greater than the fit outcome, which is attributed to material imperfections and residual surfactant at the nanowire’s surface. We experimentally extracted a propagation length of *L*_p_ ≈ 1.0 μm from laser transmission experiments, which is consistent with the fit result but also subject to large variations (see Supporting Information S7). Additionally, we find an overall scaling parameter of 1.23, indicating that all major contributions are included in the model.

The interference model in Figure 3c implies that the waveguide coupling rate is substantially larger toward the near end of the wire compared with the infinite length waveguide. The coupling rate can in principle be increased up to a factor of 4 for a reflection coefficient of *r* = 1. This would result in a coupling efficiency of 5.5%. The overall efficiency of the device could be further optimized by impedance-matching the waveguide ends^36^ and achieving constructive interference with substrate reflections.^16^

In summary, we have demonstrated the coupling of single self-assembled GaAs quantum dots to silver nanowires in the intermediate field. This is achieved by balancing coupling and propagation efficiency using a planar dielectric spacer of about 130 nm thickness. The relative positions of quantum dots and nanowires are determined with high accuracy by simultaneously imaging them through low-temperature cathodoluminescence. This enabled us to establish an interference model that explains the varying coupling efficiencies. The reflection of the propagating plasmon at the wire’s near end boosts the efficiency by up to four times. Intermediate field coupling does not necessitate nanostructuring processes in the QD’s dielectric surroundings, which often degrade the (quantum) optical characteristics of the QD. Furthermore, the intermediate field approach is not limited to QDs grown near the surface because of its weak depth dependence (see Figure 1b). Taken together, it will be possible to interface Fourier-limited quantum emitters with plasmonic structures via intermediate field coupling. An intriguing example would be an electrically pumped source of single plasmons by embedding the QDs in a diode structure.^37^

## Supporting Information Available

Definition of the coupling efficiency; sample fabrication; additional data for coupled nanosystems and methods; signal-to-background ratio; extraction of the coupling efficiency; influence of the dipole moment orientation; propagation length (PDF)
