# Supplementary material for: Intermediate Field Coupling of Single Epitaxial Quantum Dots to Plasmonic Waveguides
Source: Nano Lett. 2023 Nov 2;23(22):10532–7. doi: 10.1021/acs.nanolett.3c03442 (PMC10683061; doi:10.1021/acs.nanolett.3c03442)
Supplement: Supplementary file 1 — nl3c03442_si_001.pdf [file nl3c03442_si_001.pdf]

Supporting Information: Intermediate Field Coupling of Single Epitaxial Quantum Dots to Plasmonic Waveguides

Michael Seidel,[1] Yuhui Yang,[2] Thorsten Schumacher,[1] Yongheng Huo,[3] Saimon Filipe Covre da Silva,[3] Sven Rodt,[2] Armando Rastelli,[3] Stephan Reitzenstein,[2,*] and Markus Lippitz[1,†]

[1]*Experimental Physics III, University of Bayreuth, 95447 Bayreuth, Germany*

[2]*Institute of Solid State Physics, Technische Universität Berlin, 10623 Berlin, Germany*

[3]*Institute of Semiconductor and Solid State Physics, Johannes Kepler University Linz, Altenbergerstraße 69, A-4040 Linz, Austria*

(Dated: October 20, 2023)

S1 - DEFINITION OF THE COUPLING EFFICIENCY

Based on the framework in Ref. [1], we define the coupling efficiency

$$\eta_{in}(x,z) = \frac{3\pi c \epsilon_0 \boldsymbol{E}(x,z) \cdot \boldsymbol{E}^*(x,z)}{n_{AlGaAs}\, k_0^2 \int_A S_y \, dA} \tag{1}$$

with the free-space speed of light c, the vacuum permittivity ϵ_0, the modal electric field $\boldsymbol{E}$ in the transverse xz-plane, the refractive index $n_{AlGaAs} = 3.44$, the vacuum wavenumber k_0, and the time-averaged Poynting vector component $S_y = \frac{1}{2}\Re(\boldsymbol{E} \times \boldsymbol{H}^*) \cdot \boldsymbol{y}$ in waveguide direction y, which is integrated over the transverse plane A. The coupling efficiency η_{in} is normalized to the emission of a dipole in homogeneous AlGaAs, and therefore can exceed unity.

Furthermore, it is practical to define the normalization constant

$$p = \frac{3\pi c \epsilon_0}{n_{AlGaAs} k_0^2 \int_A S_y \, dA}, \tag{2}$$

to obtain the normalized electric field

$$\boldsymbol{E}_{norm}(x,y,z) = \frac{\boldsymbol{E}(x,y,z)}{\sqrt{p}}. \tag{3}$$

Now the coupling efficiency directly follows from

$$\eta_{in}(x,y,z) = \boldsymbol{E}_{norm}(x,y,z) \cdot \boldsymbol{E}^*_{norm}(x,y,z). \tag{4}$$

The normalized fields $\boldsymbol{E}_{norm}$ allow to evolve the coupling efficiency along the waveguide axis by combining the 2D mode analysis with Eq. 2 in the main text, as done in Fig. 3b in the main text. Considering the case $r = 0$, we find a constant coupling efficiency along the y-axis, as one would expect without reflections.

S2 - SAMPLE FABRICATION

The GaAs/AlGaAs quantum dot (QD) samples used in this work are grown after the recipe described in Ref. [2] and have also been utilized in Ref. [3, 4]. Specifically, three slightly different samples with QD burial depths $z_b = 15\,\text{nm}$, $30\,\text{nm}$ and $40\,\text{nm}$ were used. The influence of the burial depth on the waveguide coupling efficiency is negligible according to Fig. 1b in the main text.

For the dielectric spacer we use a polysiloxane-based spin-on glass (IC1-200 Intermediate Coating, Futurrex). IC1-200 is diluted in isobutanol with a 1:1 mixture. Afterwards, the IC1 is spin-coated on top of the semiconductor at 77 rps and baked out on a hotplate at 200 °C for two minutes, resulting in a film thickness of around 130 nm, which is confirmed by AFM measurements.

Finally, chemically-grown monocrystalline silver nanowires (PL-AgW100, diluted in isopropanol, PlasmaChem) are dispersed on top of the IC1 film. Afterwards, the sample is rinsed gently in ethanol in order to remove the PVP (Polyvinylpyrrolidone) surfactant, and dried in nitrogen flux. In order to avoid degradation of the Ag nanowires (NWs), the samples are coated with 5 nm of Al_2O_3 by atomic layer deposition. During transport and storing, the samples are kept under vacuum conditions.

S3 - ADDITIONAL DATA FOR COUPLED NANOSYSTEMS AND METHODS

We have collected complete datasets for nine coupled quantum dot - nanowire (QD-NW) systems. A dataset (measured at 20 K if not noted else) comprises:

- **Cathodoluminescence / Secondary electron microscope (SEM) images**
- **Photoluminescence / Reflection images via confocal laser scanning**
- **Waveguide propagation images**
- **QD and SPP emission spectra**

In Fig. S3, complete datasets for three QD-NW systems are shown, including QD emission and SPP spectra which are not shown in the main text. Tab. S3 gives an overview of the experimental quantitites extracted from these measurements. In the following, we discuss applied methods and data processing in greater detail.

Cathodoluminescence / SEM images

From the cathodoluminescence/SEM images (Fig. 2a in the main text and Fig. S3a) we extract the quantum dot positions x_{QD} and y_{QD} relative to the nanowire end, with the y-axis parallel and the x-axis perpendicular to the nanowire axis. This is done by fitting a background-corrected two-dimensional Gaussian function to the cathodoluminescence spot

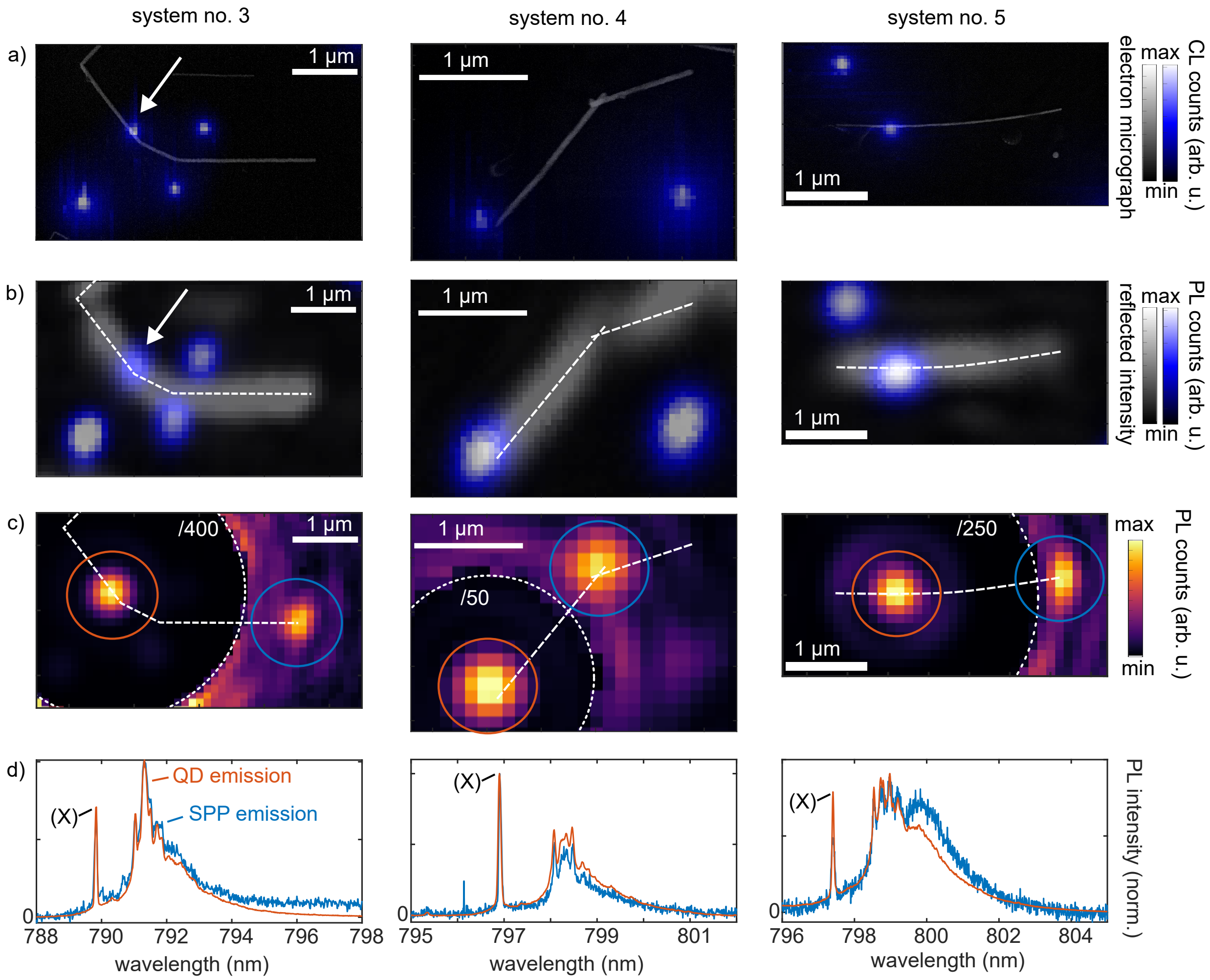

Fig. S3. **Additional data on three coupled QD-NW systems.** a) Overlaid cathodoluminescence and SEM scans. b) Overlaid photoluminescence and polarized reflection scans. c) Propagation image, demonstrating launching of SPPs. The red circle indicates the excited QD, the blue circle highlights the scattered SPP at the nanowire end. The region around the excited QD is software attenuated to increase visibility. d) Photoluminescence spectra of the QD emission and the outcoupled SPP. The excitonic emission line (X) is highlighted.

of the respective quantum dot. The uncertainties in x_{QD} (y_{QD}) shown in Fig. 3b,c in the main text are composed of the uncertainty of the fit and the determination of the nanowire center (end). As the SEM image and the corresponding cathodoluminescence map are recorded simultaneously and therefore share the same coordinates, these uncertainties are small ($< 30\,\text{nm}$).

	system no.								
	1	2	3	4	5	6	7	8	9
NW diameter (nm)	48	53	53	62	42	60	63	50	45
NW length (µm)	2.87	3.75	4.25	1.63	2.87	2.61	5.05	3.71	3.27
QD burial depth (nm)	15	30	40	40	40	30	30	30	30
IC1 thickness (nm)	131	162	136	136	136	162	162	162	162
x_{QD} (nm)	77	8	48	115	62	162	118	4	143
y_{QD} (nm)	685	593	1290	6	698	807	228	390	505
I_{spp}/I_{qd} $(\cdot 10^{-3})$	5.4	4.2	1.6	17.7	2.1	5.0	0.22	0.26	0.85
$\eta_{in,exp}$ (%)	0.66	1.68	0.49	1.21	0.24	0.41	0.63	0.13	0.22
$\eta_{in,exp,shift}$ (%)	0.85	1.68	0.54	2.18	0.29	1.34	1.19	0.13	0.55

TABLE S3. **Overview of the extracted experimental quantities for nine coupled QD-NW-systems.** Nanowire dimensions and relative QD positions (x_{QD}, y_{QD}) are obtained from cathodoluminescence/SEM images. The emission ratio I_{spp}/I_{qd} is obtained from the waveguide propagation images. For the extraction of the coupling efficiency $\eta_{in,exp}$ and $\eta_{in,exp,shift}$, see Supporting Information S5. QD burial depth and IC1 film thickness are given for the sake of completeness.

Photoluminescence / Reflection via confocal laser scanning

As mentioned in the main text, we perform two subsequent confocal laser scans and detect photoluminescence and reflection. Afterwards, both images are overlaid by transparency (Fig. 2b in the main text and Fig. S3b). For the reflection mapping, polarisation contrast is utilized to enhance the visibility of the silver nanowires. Therefore, the reflected laser ($\lambda = 635$ nm) is suppressed with an analyzer. As a result, only light which is polarized along the nanowires is collected by the APD based single-photon counting module. For a single scan, the contrast depends on the direction of the nanowires. Consequently, we add up several scans for different laser polarizations. This allows us to map the optical (photoluminescence/reflection) images to the cathodoluminescence/SEM images. Thereby, we rotate and scale the optical axes according to coordinate system given by the cathodoluminescence, which we expect to be the most accurate.

Waveguide propagation imaging

For the demonstration of QD-NW coupling, the QD is optically excited and the surrounding including the Ag nanowire is imaged onto the CCD-camera, while the excitation laser ($\lambda = 635\,\text{nm}$) is filtered out by a bandpass. The scattered SPP signal is detected together with the direct QD emission (Fig. 2c in the main text and Fig. S3c). A background-corrected two-dimensional elliptical Gaussian is fitted to the quantum dot emission and the out-coupled surface plasmon emission, respectively. Integration of the Gaussian function finally leads to the SPP-QD-emission ratio I_{spp}/I_{qd}. This emission ratio will be used to determine the coupling efficiency $\eta_{in,exp}$ for each nanosystem in Supporting Information S5.

For a subset of the investigated QD-NW systems, no clear SPP emission is observed. Instead of omitting these datasets, we decided to include these into our model (Fig. 3, main text). Just like the other systems, a 2D Gaussian is fitted at the nanowire end where the outcoupling is expected. The resulting SPP-QD emission ratio is declared as an upper limit for the true signal. Consequently, for these datapoints the uncertainty bar goes down to zero in Fig. 3c in the main text. As can be seen in the same figure, the x_{QD}-corrected coupling efficiency of these datapoints is consistent with our model. In other words, the vanishing emission is explained by a small coupling efficiency at the respective QD position, either due to large lateral offset from the wire axis, or destructive interference of the SPPs.

QD and SPP emission spectra

The spectra of the direct QD photoluminescence and the scattered SPP shown in Fig. S3d are recorded by spatial filtering before entering the entrance slit of the spectrometer. We find identical spectra for direct QD emission and remote SPP emission. There is no influence of the sample treatment, for example the cathdoluminescence measurements, on the spectral characteristics of the quantum dots observable. The linewidths of the corresponding excitonic transitions (X) in the photoluminescence spectra are around 60-80 µeV. Such values are expected for near-surface grown GaAs quantum dots due to interaction of confined excitons with surface states, see for example Ref. [3]. In this work, it is also shown that the red-shifted multi-excitonic transitions tend to broaden for thinner capping layers. This broadening is also apparent in the photoluminescence spectra shown in Fig. S3d.

S4 - SIGNAL-TO-BACKGROUND RATIO

For most of the nanosystems, emission is observed only from the far wire end. For the near wire end, which typically is in a distance below 1 µm from the QD, the SPP emission competes with a much stronger airy-patterned background of the direct QD emission, as shown in Fig. S4a. Here, the intensity cross section of an uncoupled QD emission image is fitted with an airy function. We compare the QD intensity distribution with an exponential function which represents the expected SPP emission intensity (following Eq. 7, see Supporting Information S5)

$$I_{spp}(y) = I_{qd}(0)\,\eta_{in}(1-|r|^2)\frac{\eta_{spp,ff}}{\eta_{qd,ff}}e^{-y/L_p} \tag{5}$$

as a function of the distance y from the QD. Here, the QD intensity $I_{qd}(0) = 1$ is normalized and the other parameters $r = 0.65$, $L_p = 0.86\,\mu m$ and $\frac{\eta_{spp,ff}}{\eta_{qd,ff}} = 17$ are taken as in the main text. For the coupling efficiency η_{in}, we use the values for the nanosystems with highest and lowest efficiency according to Tab. S3, respectively. It can be seen that for small distances, the airy-patterned background dominates, matching our observations. In this simple picture, different coupling efficiencies shift the exponential function against the background.

These findings are supported by nanosystem no. 2, featuring the highest coupling efficiency (see Tab. S3) and signal-to-background ratio. Fig. S4b shows the QD-NW coupling image for this nanosystem with SPP emission from the short wire end (SPP, 1), overlapping with the QD's airy pattern (QD). At the far wire end (SPP, 2), the SPP-signal to QD-background ratio is much higher. Another short wire does not show any SPP emission, which can be explained by the larger distance from the QD and the large angle with the longer wire.

S5 - EXTRACTION OF THE COUPLING EFFICIENCY

We write the detected intensity at the QD (I_{qd}) or waveguide end (I_{spp}) as product of the respective efficiencies, starting from the same total QD emission I_0. For the direct QD signal, only the far-field collection efficiency $\eta_{qd,ff}$ enters. For the waveguide, we need to take into account the coupling efficiency η_{in} and the plasmon propagation length. Moreover, the finite reflection r at the waveguide end and the far-field collection efficiency $\eta_{spp,ff}$ need to be considered.

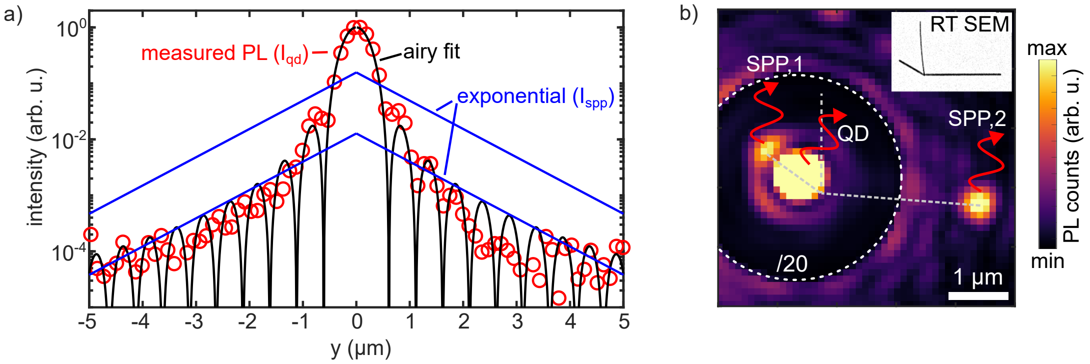

Fig. S4. **Dependence of the SPP-emission to QD-background on the length of the nanowire.** a) Measured (red circles) and fitted (black line) intensity cross section of an uncoupled QD emission, imaged onto the CCD-camera. For comparison, the blue lines represent the expected SPP emission intensities for the nanosystems with the highest and lowest coupling efficiency as function of the distance from the emitter at $y = 0$. b) QD-NW coupling image of nanosystem no. 2. Clear SPP emission is observable at the far wire end (SPP, 2), while the SPP emission at the near wire end (SPP, 1) is overlapping with the QD's airy pattern (QD). Another short wire does not show SPP emission. Inset: room-temperature SEM image of the same nanosystem.

All together we get

$$\frac{I_{spp}}{I_{qd}} = \frac{I_0 \eta_{in} \eta_p (1 - |r|^2) \eta_{spp,ff}}{I_0 \eta_{qd,ff}} \tag{6}$$

with the field reflection amplitude r of the wire end, effectively reducing the number of out-coupled photons. Furthermore, we account for different far-field collection efficiencies for the surface plasmon $\eta_{spp,ff}$ and the quantum dot $\eta_{qd,ff}$. This leads to the experimental coupling efficiency

$$\eta_{in,exp} = \frac{I_{spp}/I_{qd}}{e^{-L/L_p}(1 - |r|^2)\frac{\eta_{spp,ff}}{\eta_{qd,ff}}}. \tag{7}$$

The QD-SPP emission ratio is extracted from the waveguide propagation images, as discussed in Supporting Information S3. The propagation losses are corrected for the length L, defined as the distance from the QD to the far wire end, which is known from the cathodoluminescence/SEM images. The propagation length L_p is a free parameter in the interference model (Fig. 3, main text), where it is determined to $L_p = 0.86\,\mu\text{m}$. This leaves the wire end reflection amplitude r and the far-field collection efficiencies $\eta_{spp,ff}$ and $\eta_{qd,ff}$, which will be derived from simulations in the following.

Complex reflection coefficient

In order to determine the complex reflection coefficient $\tilde{r} = re^{i\phi_r}$ for our system, a 3D finite element simulation (Comsol Multiphysics) is performed. The cross section of the 3D model is identical to the geometry shown in Fig. 1b in the main text, but the nanowire is terminated before reaching the end of the computation window, as sketched in Fig. S5a. We take the mode profile in the xz-plane and excite the 3D model via the port function in Comsol to obtain the electric field distribution, which is evaluated in a centered linecut at $z_b = 30\,\text{nm}$ (with respect to the nanowire axis). Now, we fit the analytical intensity distribution for the semi-infinite wire

$$|E(y)|^2 = \left| E_0 \left[e^{i\tilde{\beta}y} + e^{i\tilde{\beta}(y_0 - y)} \tilde{r} e^{i\tilde{\beta}y_0} \right] \right|^2 \tag{8}$$

to the simulated intensity, featuring the expected interference fringes (Fig. S5b). Here, E_0 is the initial mode amplitude, y the spatial coordinate along the propagation direction, $y_0 = 3\,\mu\text{m}$ the position of the wire termination, and $\tilde{\beta} = k_0 \tilde{n}_{\text{eff}}$ the complex propagation constant with the effective mode index $n_{\text{eff}} = 1.68 + 0.023i$ from the 2D mode analysis. We obtain the reflection amplitude $r = 0.647$ and reflection phase $\phi_r = -1.89$.

Collection efficiency

We expect the far-field collection efficiencies for the scattered SPP and the direct QD emission to differ significantly, since the QD mostly radiates into the high-index GaAs substrate. Therefore, 3D finite element methods simulations are performed, approximating the QD as well as the out-coupled SPP as dipolar emitters, which are located 30 nm below and 155 nm above the semiconductor surface, respectively. The thickness of the spacer layer and the refractive indices are the same as in the 2D simulation in Fig. 1b. We use the RETOP package [5] to obtain the amount of power which is radiated into the upper halfspace for the respective dipole positions. Normalized to the total emitted power, we find $\eta_{qd,ff} = 0.026$ and $\eta_{spp,ff} = 0.434$, leading to a far-field collection efficiency ratio $\frac{\eta_{spp,ff}}{\eta_{qd,ff}} \approx 17$.

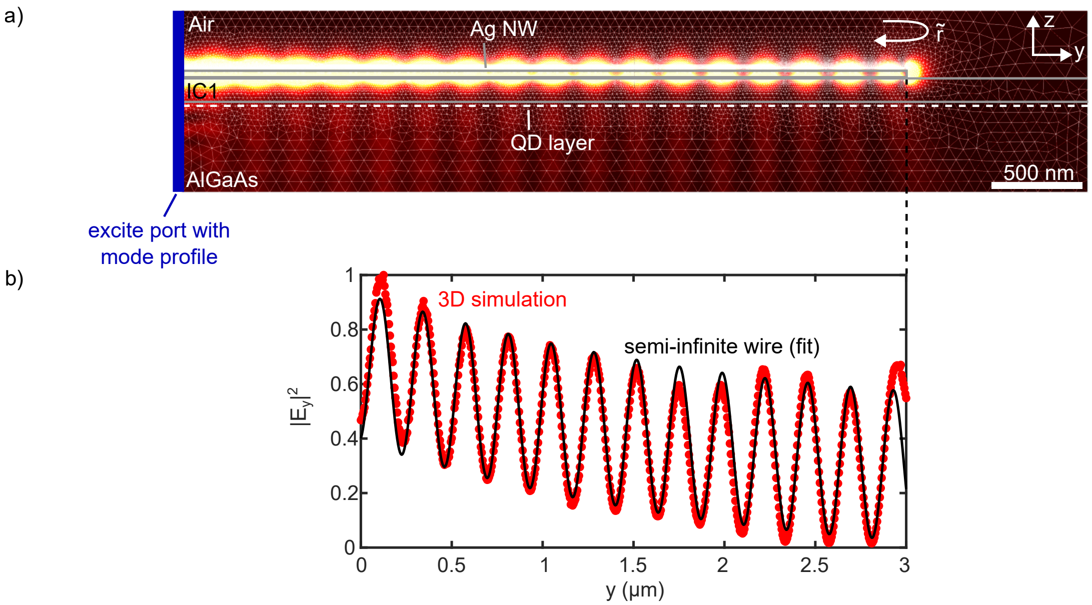

Fig. S5. **Extraction of the complex reflection coefficient for our geometry.** a) A 3D model including a terminated nanowire is excited with the mode profile from Fig. 1b in the main text. The structure is shown from the side. b) Comparison of analytic model (black line) and numerical model (red dots). The 3D port simulation is evaluated at a centered linecut at the QD layer in a depth $z_b = 30\,\text{nm}$.

S6 - INFLUENCE OF THE DIPOLE MOMENT ORIENTATION

The general expression for the coupling efficiency of an emitter with dipole moment $\boldsymbol{\mu}$ in the modal field $\boldsymbol{E}$ of the waveguide is given by

$$\eta_{in} = |\boldsymbol{\mu} \cdot \boldsymbol{E}|^2, \tag{9}$$

assuming normalized electric fields. Our epitaxial GaAs quantum dots feature two orthogonal excitonic states which both can be excited non-resonantly with our laser at $\lambda = 635\,\text{nm}$. We assume that the corresponding dipole moment contributions $\boldsymbol{\mu}_1$ and $\boldsymbol{\mu}_2$ add up incoherently and the overall incoupling efficiency can be written as

$$\eta_{in} = |\boldsymbol{\mu}_1 \cdot \boldsymbol{E}|^2 + |\boldsymbol{\mu}_2 \cdot \boldsymbol{E}|^2. \tag{10}$$

As the out-of-plane transition dipole moment $\mu_z = 0$ is vanishing, we obtain

$$\eta_{in} = |\mu_{1,x}E_x + \mu_{1,y}E_y|^2 + |\mu_{2,x}E_x + \mu_{2,y}E_y|^2. \tag{11}$$

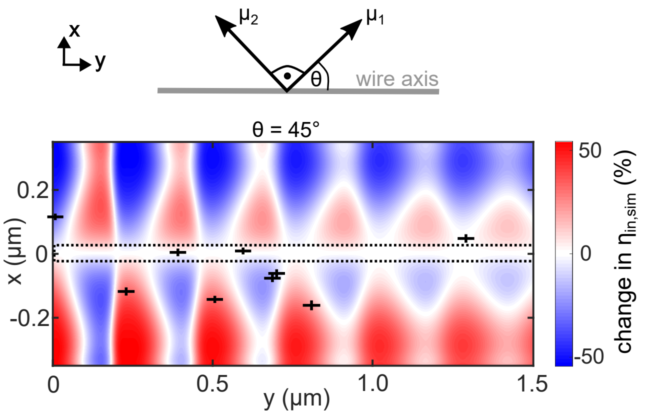

Fig. S6. **Influence of the dipole moment orientation on the coupling efficiency.** Spatially resolved (relative) change in coupling efficiency for a hypothetical angle of $\theta = 45°$. The black crosses indicate the QD positions and the dashed lines the width of the nanowire.

Assuming $|\boldsymbol{\mu}_1| = |\boldsymbol{\mu}_2|$ and exploiting the orthogonality of the excitonic states leads to

$$\eta_{in} = |\mu \sin\theta E_x + \mu \cos\theta E_y|^2 + |\mu \cos\theta E_x + \mu \sin\theta E_y|^2, \tag{12}$$

with θ being the angle between dipole moment contribution $\boldsymbol{\mu}_1$ and wire axis. As the electric field is complex-valued, we use $|a+b|^2 = |a|^2 + |b|^2 + 2\,\Re(ab^*)$ to finally obtain

$$\eta_{in} = \mu^2 \left[|E_x|^2 + |E_y|^2 + 4 \sin\theta \cos\theta\, \Re(E_x E_y^*) \right]. \tag{13}$$

The crossterm describes the influence of the dipole moment orientation on the coupling efficiency and vanishes for angles $\theta = 0°$ and $\theta = 90°$, representing parallel and perpendicular orientation with respect to the nanowire, as assumed in the main text. The spatial dependence of the modification in the coupling efficiency is shown in Fig. S6 for $\theta = 45°$, where the crossterm in Eq. 13 has its maximum. It can be seen that the dipole moment orientation is negligible close to the nanowire axis at $x = 0$, where the electric field points in the propagation direction and the E_x-component is zero. For QDs far away from the nanowire axis, the coupling efficiency can be modified up to $\pm 30\,\%$ at $\theta = 45°$. As the sign of the electric field is unkown, we take the absolute value of this hypothetical "worst case" modification as an additional uncertainty for the coupling efficiency of each QD in Fig. 3c in the main text.

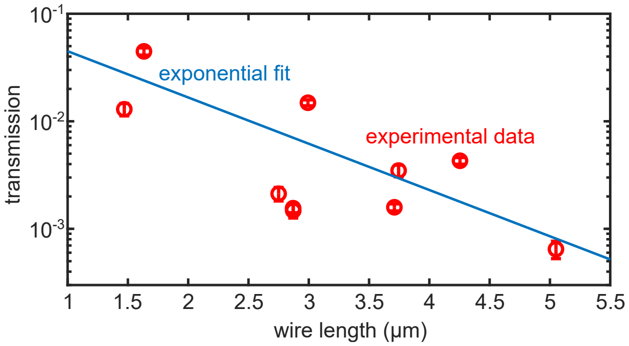

Fig. S7. **Laser transmission for several Ag nanowires to determine the surface plasmon propagation length.** Red circles: measured SPP transmission. Blue line: exponential fit, suggesting a propagation length of $L_p \approx 1.0\,\mu\text{m}$.

S7 - PROPAGATION LENGTH

In order to determine the SPP propagation length, a modelocked Titanium-Sapphire laser is operated at a wavelength $\lambda = 795\,\text{nm}$ to match the QD emission wavelength. The laser is polarized parallel with respect to the nanowire axis and focused on one of its ends. The direct laser reflection as well as the out-coupled SPP emission at the other wire end is imaged onto the CCD-camera. In order to obtain the transmission (see Fig. S7), the SPP emission is normalized to the laser reflection. The wire lengths are taken from SEM images, respectively. By fitting an exponential function on the transmission data, we find a $1/e$-propagation length $L_p \approx 1.0\,\mu\text{m}$, consistent with the fit result in the main text. It must be stated that the spread of the data points is rather large, leading to a large uncertainty of the fit. One reason for that may be that the laser coupling is sensitive to the exact shape of the laser focus as well as lateral position regarding the nanowire end. In contrast, for the QD coupling experiment, the exact focusing conditions are less sensitive, as the laser only generates electron-hole pairs in the environment of the QD.

---

* stephan.reitzenstein@physik.tu-berlin.de

† markus.lippitz@uni-bayreuth.de

[1] Chen, Y.; Nielsen, T. R.; Gregersen, N.; Lodahl, P.; Mørk, J. Finite-element modeling of spontaneous emission of a quantum emitter at nanoscale proximity to plasmonic waveguides.

Physical Review B - Condensed Matter and Materials Physics **2010**, *81*, 125431.

[2] Huo, Y. H.; Rastelli, A.; Schmidt, O. G. Ultra-small excitonic fine structure splitting in highly symmetric quantum dots on GaAs (001) substrate. *Applied Physics Letters* **2013**, *102*, 152105.

[3] Zhang, H.; Huo, Y.; Lindfors, K.; Chen, Y.; Schmidt, O. G.; Rastelli, A.; Lippitz, M. Narrow-line self-assembled GaAs quantum dots for plasmonics. *Applied Physics Letters* **2015**, *106*, 101110.

[4] Wu, X.; Jiang, P.; Razinskas, G.; Huo, Y.; Zhang, H.; Kamp, M.; Rastelli, A.; Schmidt, O. G.; Hecht, B.; Lindfors, K.; Lippitz, M. On-Chip Single-Plasmon Nanocircuit Driven by a Self-Assembled Quantum Dot. *Nano Letters* **2017**, *17*, 4291–4296.

[5] Yang, J.; Hugonin, J. P.; Lalanne, P. Near-to-Far Field Transformations for Radiative and Guided Waves. *ACS Photonics* **2016**, *3*, 395–402.
